# Whole genome sequence data of *Aeromonas diversa* SAU1, isolated from Tilapia (*Oreochromis niloticus*)

**DOI:** 10.1016/j.dib.2025.111396

**Published:** 2025-02-17

**Authors:** Sayed Mashequl Bari, Ishtiaque Ahammad, Arittra Bhattacharjee, A.M. Shahabuddin

**Affiliations:** a Department of Aquatic Animal Health Management, Sher-e-Bangla Agricultural University, Dhaka 1207, Bangladesh; b Bioinformatics Division, National Institute of Biotechnology, Dhaka 1349, Bangladesh; c Department of Aquaculture, Sher-e-Bangla Agricultural University, Dhaka 1207, Bangladesh

**Keywords:** Whole genome sequence, *Aeromonas diversa*, Tilapia, Fish, Illumina, Antimicrobial resistance, Virulence genes

## Abstract

*Aeromonas diversa* SAU1 was isolated from Tilapia (*Oreochromis niloticus*) that was cultured in a Recirculating Aquaculture System (RAS) in Bangladesh. The genome was sequenced using the Illumina MiSeq platform and assembled using the de novo genome assembly method. The draft genome was annotated and virulence factors, antimicrobial resistance related genes, CRISPER-Cas systems, metabolic pathways, and biosynthetic gene clusters were predicted. The genome is approximately 4.1 mb in size. The assembled genome comprises 4,105,655 bp with GC content of 61.63 %, which includes 3,823 protein-coding genes and 25 pseudogenes. Identifying the characteristics of the *A. diversa* genome can help us understand its harmful effects and analyze the biocontrol mechanism in aquaculture systems.

Specifications Table

**Table:** 

Subject	Microbiology
Specific subject area	Molecular biology, genomics and aquaculture
Type of data	Raw sequencing data, assembled and annotated draft genome
Data collection	The bacterial sample was isolated from infected tilapia (*O. niloticus*). Bacterial DNA was extracted using the Qiagen bacterial genomic DNA extraction kit. The DNA was then sent to Wuhan Tianyi Huayu Gene Technology Co., Ltd., China for genome sequencing using the Illumina MiSeq platform. The sequenced data were trimmed, assembled, and annotated using Fatsp, Unicycler, and the NCBI Prokaryotic Genome Annotation Pipeline (PGAP), respectively*.*
Data source location	Institution: Department of Aquatic Animal Health Management, Sher-e-Bangla Agricultural University City: Dhaka Country: Bangladesh Coordinates: 23°46′18.8″N 90°22′19.3″E*.*
Data accessibility	Repository name: National Centre for Biotechnology Information (NCBI) Data identification number: GenBank: JBFMMD000000000; BioProject: PRJNA1135548; BioSample: SAMN42485447; Sequence Read Archive (SRA): SRR29825452 Direct URL to data: GenBank https://www.ncbi.nlm.nih.gov/datasets/genome/GCA_040790265.1/ Bio project: https://www.ncbi.nlm.nih.gov/bioproject/?term=PRJNA1135548 Bio sample: https://www.ncbi.nlm.nih.gov/biosample/?term=SAMN42485447 SRA: https://www.ncbi.nlm.nih.gov/sra?LinkName=biosample_sra&from_uid=42485447
Related research article	Not Applicable

## 1 Value of the Data

• The data is valuable for fish disease management by providing insights into the genome structure of *A. diversa* SAU1 isolated from a commercial aquaculture system.
• The antibiotic resistance and virulence profiling of the *A. diversa* SAU1 strain are essential for evaluating targeted treatment challenges in aquaculture and aiding in the development of effective antimicrobial strategies.
• The genome sequence of *A. diversa* SAU1 offers key insights for biotechnological applications in aquaculture and supports comparative genomics analysis.

## 2 Background

Motile Aeromonas Septicemia (MAS), caused by *Aeromonas* spp., is a widespread infectious disease found in freshwater fish species across Southeast Asia [1]. *A. diversa* is a gram-negative, facultative anaerobic bacterium that can cause significant infections in fish [2,3]. This pathogen is less frequently reported [4] than other *Aeromonas* species [5] such as *A. hydrophila, A. veronii, A. salmonicida* and others but it may pose a notable threat in aquaculture systems. In 2023, *A. diversa* SAU1 was isolated from a diseased tilapia (*O. niloticus*) that exhibited clinical sign of hemorrhages on the skin and fins, exophthalmia, and abdominal distension in a Recirculating Aquaculture System (RAS) at Sher-e-Bangla Agricultural University in Dhaka, Bangladesh.

## 3 Data Description

**Fig. 1:**
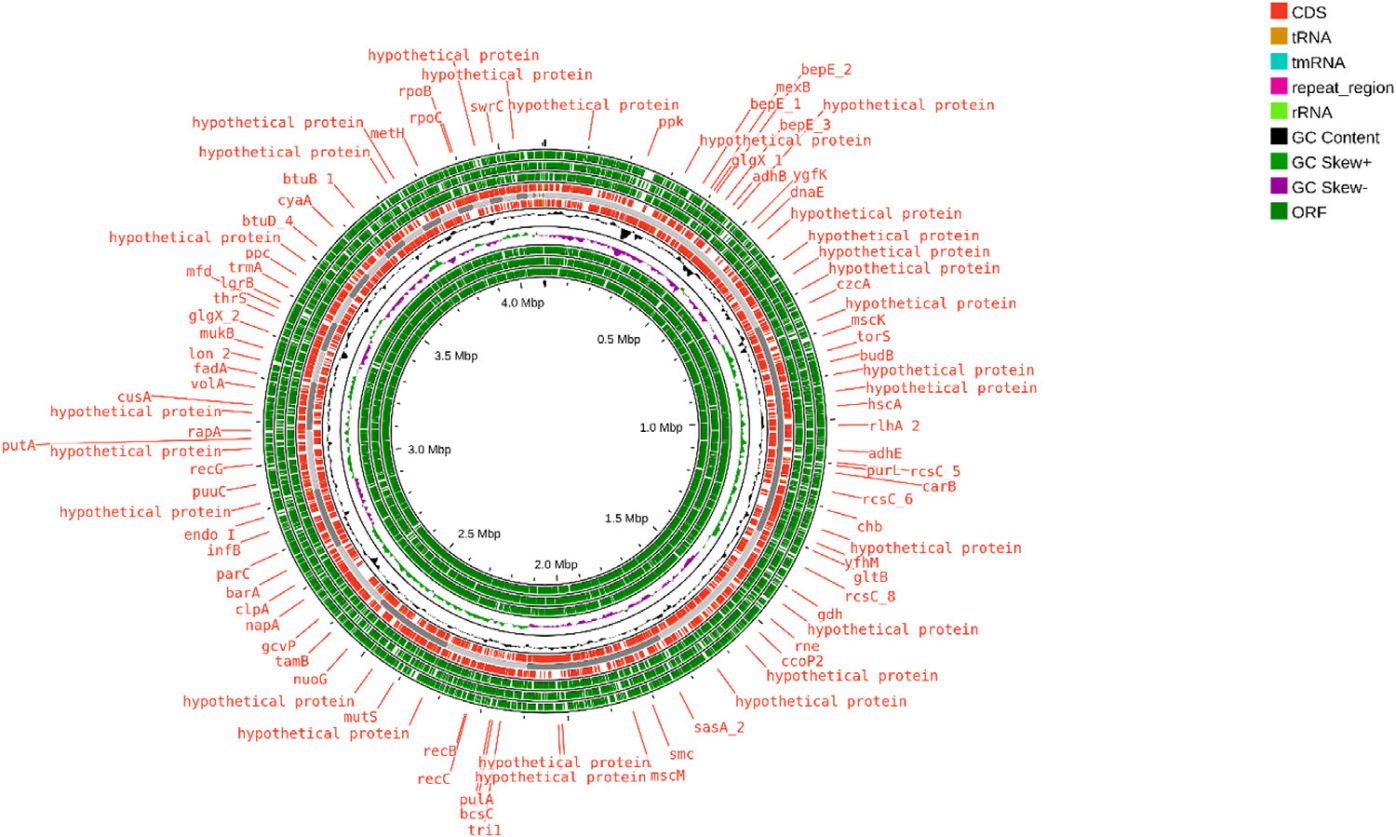
Circular visualization of *A. diversa* SAU1 genome generated using Proksee (https://proksee.ca/). The genomic features are depicted through circular tracks (from outside to inside). The outermost 3 tracks (Track 1, 2, and 3) depict 3 open reading frames (ORFs). Track 4 depicts coding sequences (CDS), tRNA, rRNA, tmRNA, and repeat regions. Track 5 and track 6 depict the GC content and the GC skew respectively. The innermost 3 tracks (Track 7, 8, and 9) depict the rest of the ORFs. The genome size: 4105,655bp.

In this study, we present the whole-genome sequencing data for *A. diversa* SAU1. Additionally, we conduct a comprehensive analysis of its genome data, which includes taxonomic identification, assessment of antimicrobial resistance, detection of virulence factors, analysis of metabolic pathways, and evaluation of the CRISPR array. The raw paired end sequencing data generated by Illumina MiSeq platform provided two fastq files each containing 1920,723,750 base pairs. The assembled genome contains 33 contigs with an overall coverage of 945.5×. The genome has a total length of 4105,655 bp with a G + C content of 61.5 %. The total number of reads produced was 3841,447,500 bp with a median contig length (N50) of 384,764 bp, a largest contig of 729,309 bp, and a shortest contig of 1235 bp. Genome annotation predicted 3945 genes, 3823 of which are protein-coding genes. Additionally, 122 non-coding RNA (ncRNA) genes were identified, including 3 ribosomal RNAs (rRNAs), 87 transfer RNAs (tRNAs), and 25 pseudogenes. Three antibiotic-resistant gene homologs were identified in the genome: ampH_1, sul2_2, and tet(A)_4. The ampH_1 gene is associated with resistance to beta-lactam antibiotics, sul2_2 confers resistance to sulfonamide, and tet(A)_4 provides resistance to tetracycline. A virulent gene pscR was also identified, which encoding translocation protein in type III secretion. One CRISPR spacer was identified, providing insight into the genomeʼs adaptive immune system. Eight metabolic pathway modules were identified with complete coverage (100 %), and no biosynthetic gene clusters were found (Fig. 1), (Table 1).

**Table 1:** Genome statistic and features of *A. diversa* SAU1.

Parameter	Value
BioProject no.	PRJNA1135548
BioSample no.	SAMN42485447
Illumina reads accession no.	SRR29825452
Genome assembly accession no.	JBFMMD000000000
No. of Illumina reads	25,609,650
Total no of Illumina bases	3,841,447,500
Average genome coverage	945.5
Genome completeness	99.5 %
Genome size	4.1 mb
No. of Contigs	33
N50 value	384,764
L50 value	4
No. of Bases	4,105,655
Read Length	150
Total no of Gene	3945
No. of protein-coding Gene	3823
No. of rRNA	3
No. of tRNA	87
Pseudogenes	25
GC content (%)	61.63
Shortest contig size	1235
Median sequence size	60,577
Mean sequence size	124,413.8
Longest contig size	729,309

## 4 Experimental Design, Materials and Methods

**Fig. 2:**
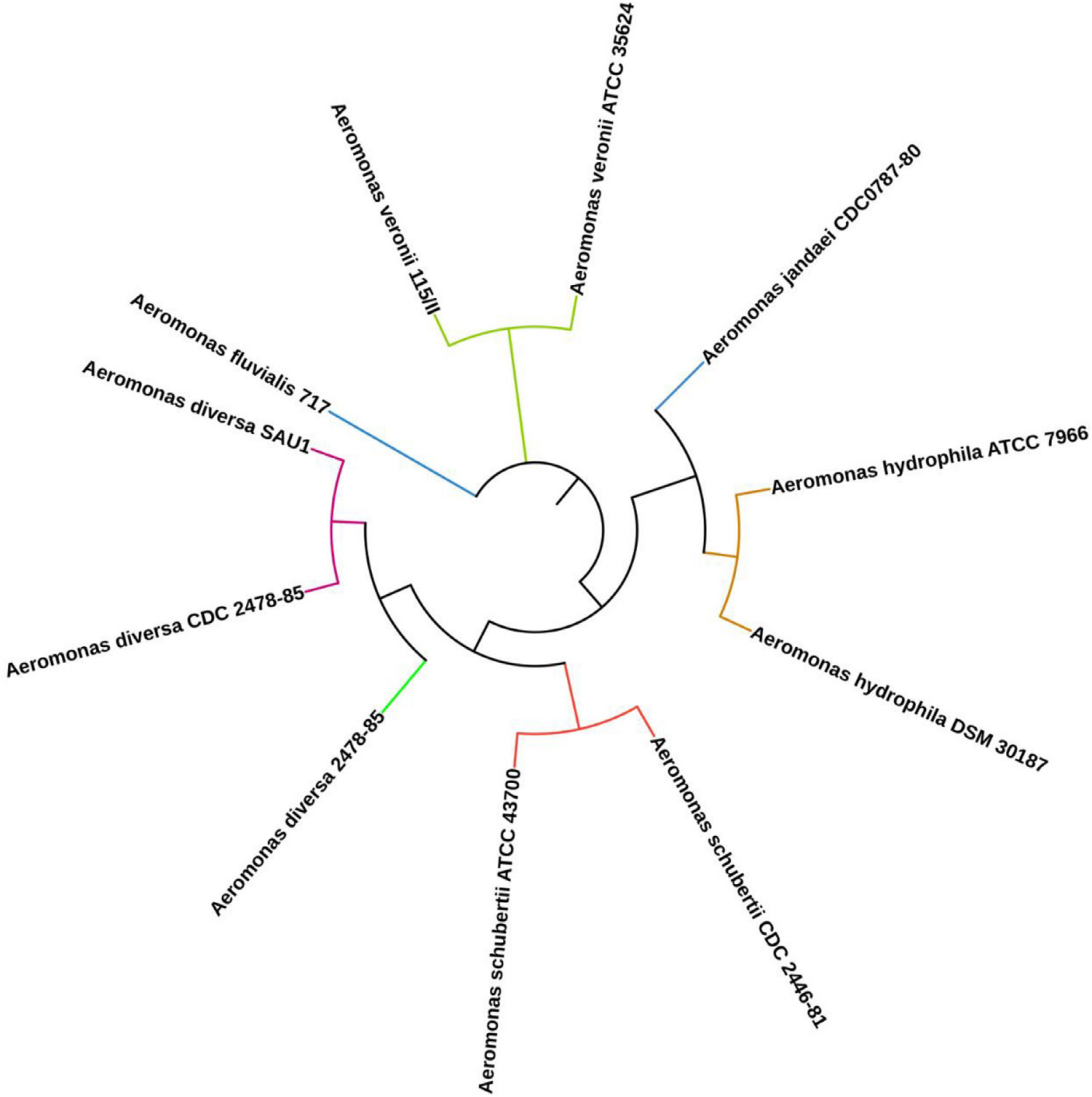
16s *rRNA* gene-based phylogenetic tree of various *Aeromonas* species including *A. diversa SAU1* visualized using iTOL (https://itol.embl.de/)*.* The different colored branch nodes signify different clades.

The sampled fish were handled according to the guidelines of the Sher-e-Bangla Agricultural University research ethics committee. Forty-five infected tilapia exhibiting clinical signs of exophthalmia, hemorrhages, and abdominal distension were aseptically collected from eight Recirculatory Aquaculture System (RAS) tanks at Sher-e-Bangla Agricultural University and transferred to the Fish Disease Laboratory for bacterial identification. Infected tissue and abdominal fluid samples were cultured on MacConkey agar at 37 °C for 24 h and then sub-cultured on blood agar at 37 °C for another 24 h to isolate pure colonies. Total DNA was extracted from a pure colony using the DNeasy Blood & Tissue Kit (Qiagen, Germany), following the manufacturerʼs protocol. The genomic DNA was qualitatively and quantitatively assessed using a NanoDrop 2000 UV–visible spectrophotometer (Thermo Fisher Scientific). Wuhan Tianyi Huayu Gene Technology Co., Ltd., China sequenced the genome using the Illumina MiSeq platform with paired-end (150 × 2) library preparation. The sequence reads were trimmed with Fastp (version 0.23.2) [6] to remove low-quality bases and adapter sequences, ensuring high-quality data for analysis. The trimmed reads were used for de novo assembly with Unicycler (version 0.4.8) [7]. The comprehensive and reliable genomic map obtained from this approach is crucial for further genetic and functional analysis. Gene prediction and functional annotation were performed using the NCBI Prokaryotic Genome Annotation Pipeline (PGAP) (version 6.7) [8]. This analysis provided detailed insights into the genomic structure and facilitated further genetic and biological analyses. Antibiotic resistance genes were identified using Abricate (version 0.5) [9], which confirmed resistance to specific antibiotics based on the ResFinder 4.5 database [10]. This analysis provided critical insights into the genetic basis of antibiotic resistance. Virulence genes were identified using the Virulence Factor Database (VFDB) [11], which helped elucidate the pathogenic mechanisms of the *A. diversa* SAU1 strain. Metabolic pathways were detected with MicrobeAnnotator (version 2.0.5) [12]. Secondary metabolite biosynthetic gene clusters (SM-BGCs) were predicted using 'antibiotics & Secondary Metabolite Analysis Shell' (antiSMASH) (version 6) [13]. CRISPR-Cas identification was performed using CRISPRCasTyper (version 1.4.1) [14], which facilitated the understanding of the strain's adaptive immune mechanisms, including its potential to target and neutralize foreign DNA elements*.* The circular plot of the genome was generated using Proksee (version 1.0.0a6) [15]. The 16s rRNA gene of the *A. diversa* SAU1 strain was subjected to National Center for Biotechnology Information Basic Local Alignment Tool (NCBI-BLAST) (version 2.16.0) to retrieve closely related gene sequences [16]. Afterward, the sequences were aligned using Multiple Alignment using Fast Fourier Transform (MAFFT) (version 7.525) and a phylogenetic tree was construed using Unweighted Pair Group Method with Arithmetic Mean (UPGMA) method [17]. The tree was visualized via the Interactive Tree of Life (iTOL) (Fig. 2) (version 6) [18]. Identification and taxonomic classification of *A. diversa* SAU1 was carried out using average nucleotide identity (ANI) via JSpeciesWS (https://jspecies.ribohost.com/jspeciesws/) (Table 2). All software/tools used in this study were run with default parameters.

**Table 2:** Average nucleotide identity (ANI) between several *Aeromonas* species.

	*Aeromonas diversa* CDC 2478-85	*Aeromonas diversa* SAU1	*Aeromonas fluvialis*	*Aeromonas hydrophila*	*Aeromonas jandaei*	*Aeromonas schubertii*	*Aeromonas veronii*
*Aeromonas diversa* CDC 2478-85	100	98.8	77.38	77.1	77.52	88.8	77.29
*Aeromonas diversa* SAU1	98.83	100	77.41	77.63	77.65	88.76	77.43
*Aeromonas fluvialis*	77.62	77.65	100	82.74	87.73	77.75	91.77
*Aeromonas hydrophila*	80.87	80.96	87.34	100	87.63	81.09	86.88
*Aeromonas jandaei*	77.6	77.56	87.45	86.11	100	77.45	88.8
*Aeromonas schubertii*	88.87	88.77	77.78	78.11	77.77	100	77.64
*Aeromonas veronii*	77.59	77.66	91.43	86.22	88.76	77.51	100

## Limitations

None

## Ethics Statement

This is an observational study. The Sher-e-Bangla Agricultural University research ethics committee has confirmed that no ethical approval is required.

## CRediT authorship contribution statement

**Sayed Mashequl Bari:** Conceptualization, Methodology, Data curation, Investigation, Formal analysis, Visualization, Writing – original draft. **Ishtiaque Ahammad:** Formal analysis. **Arittra Bhattacharjee:** Formal analysis. **A.M. Shahabuddin:** Writing – review & editing.

## Data Availability

NCBIWhole genome sequence data (Original data)
